# Six-sigma and quality planning of TORCH tests in the Peruvian population: a single-center cross-sectional study

**DOI:** 10.1186/s13104-022-05904-9

**Published:** 2022-01-11

**Authors:** Jeel Moya-Salazar, Bianca M. SantaMaria, Marcia M. Moya-Salazar, Víctor Rojas-Zumaran, Karina Chicoma-Flores, Hans Contreras-Pulache

**Affiliations:** 1 Department of Pathology, Hospital Nacional Docente Madre-Niño San Bartolomé, Lima, Peru; 2 Faculties of Health Science, School of Medicine, Universidad Norbert Wiener, 444 Arequipa Av., 51001 Lima, Peru; 3 Faculties of Health Science, School of Medical Technology, Universidad Norbert Wiener, Lima, Peru; 4 Clinical and Laboratory Department, Suiza Lab, Lima, Peru; 5 Faculty of Pharmacy and Biochemistry, Universidad Norbert Wiener, Lima, Peru; 6 South America Center for Education and Research in Public Health, Universidad Norbert Wiener, Lima, Peru

**Keywords:** Six sigma, Toxoplasma, Quality assurance, Cytomegalovirus, Quality management, Enzyme-linked immunosorbent assay

## Abstract

**Objective:**

To ensure the health of newborns, it is necessary to perform high-quality diagnostic tests. The TORCH panel is a set of tests that identifies infectious pathogens such as Toxoplasma (Toxo) and Cytomegalovirus (CMV) that are common in low-setting populations. We performed TORCH panel quality planning using six sigma in a reference laboratory at Peru.

**Results:**

This was a cross-sectional study. TORCH tests include Toxo, Rubella, CMV, and Herpes. We processed all samples by fourth-generation ELISA on the GEMINI XCR200 analyzer (Diatron, Budapest, Hungary). We obtained the imprecision from the annual data of the external quality assessment plan and we used the CLSI EP12-A3 guideline. In a total of 44,788 analyses, the average imprecision was 3.69 ± 1.47%, and CMV had lower imprecision (2.3 and 2.6% for IgM and IgG, respectively). Quality planning of the TORCH panel allowed estimating the sigma value that ranged from 4 to 10 (average 7 ± 2 sigma), where rubella had the highest values (10 for IgM and 8 for IgG) while HSV2 had the lowest values (4 for IgM and 5 for IgG). Our results suggest the optimal performance of half of the markers including Toxoplasma, Rubella, and CMV in the Peruvian population.

## Introduction

One of the main threats to neonatal and child health is the high rates of infections that cause 1.2 million deaths each year [1]. Therefore, one of the main guidelines of the Sustainable Development Goals for 2030 is to ensure neonatal health worldwide [2]. Consequently, it is necessary to prevent infectious diseases, one of the most frequent diseases affecting the children and neonates by improving detection and follow-up systems.

Globally, perinatal management is crucial to reduce neonatal morbidity and mortality levels, mainly in low-income settings where sepsis is a common complication and infectious diseases reach their peak [3]. TORCH tests, an immunological screening panel to detect the main infectious agents associated with neonatal diseases, are used to diagnose infections [4]. The TORCH panel can be performed with immunological methods such as immunochromatography, enzyme-linked immunosorbent assay (ELISA) or chemiluminescence immunoassay (CLIA) that show the presence of antibodies against TORCH infectious agents.

ELISA tests are the most widely used methods and probably have limitations since they cannot detect significant losses (analytical sensitivity) and may interfere with the final result causing false negatives [5]. For those reasons, it is necessary to develop verification studies and quality planning in qualitative immunological methods, estimating their suitability through the six sigma or OPSpecs charts as previously demonstrated [6–8]. In Peru, many clinical laboratories have limited access to quality management processes, performing few quality assessments in qualitative tests such as ELISA tests, despite the studies that propose quality assessment schemes for ELISA [5].

In this cross-sectional study, we aim to perform TORCH panel quality planning using six sigma in Peru. With fewer studies focusing on developing quality assessments in qualitative tests, our purpose is to know the level of performance and compliance with quality requirements.

## Main text

### Study design and TORCH panel

We carried out this observational cross-sectional and single-centred study at the Suiza Lab in Lima, Peru. According to the objectives of this study we used TORCH tests for Toxoplasma, Rubella, Cytomegalovirus, and Herpes (all from Diatron, Budapest, Hungary). For all infectious markers, IgG and IgM were determined. Following the manufacturer’s specifications, the GEMINI XCR200 automated kit (Diatron, Budapest, Hungary) processed the samples by a fourth-generation qualitative ELISA. This equipment has a photometric reading range of 0–3.0 absorbance (Abs) (range 400–700 nm), an accuracy of 1% CV at 1.0 Abs, and linearity of 0–2000 Abs. The incubator features an integral wash system with a capacity of three washes and an accuracy of 10% CV at 300 μl.

### Quality analytic requirements

For each enzyme-linked immunosorbent assay, we verified calibration using linearity as previously described [6]. Data were collected for 2019 to determine the minimum acceptable concentrations measured in Abs for each marker following previous protocols [5, 6]. We assessed the correlation between the absorbance of the samples and the cut-off for each test, following the recommendations of the CLSI EP12-A3 guideline [9]. The cut-off was the measure derived from the results of three negative calibrators plus a fixed value. From this correlation, we estimated the quality requirements for each marker.

### Quality planning and data analysis

We developed quality planning according to the previous steps of defining the quality requirements. The bias was not evaluated and had a value of 0 for all the TORCH panels [10]. The imprecision of each test was estimated from the annual evaluation values of the External Quality Assessment Program in which Suiza Lab participates. From these data established for each marker, the sigma value of each test was estimated using the formula:

$$ Sigma = \frac{Quality \, requirement - bias }{{imprecision}} $$

The QC candidate selection procedure was performed under the Westgard rules: 12.5s, 13s, 13.5s, 22.5s, 13s/22s, 13.5s/22.5s y 13.5s/22.5s/R.4s [5, 11]. The sigma metric analysis was created in SPSS version 25.0 (IBM, Armonk, USA) for Windows. The estimation of the statistical control rules according to the sigma value was performed using the sigma charts following previous recommendations [12]. This selection was developed considering the best fit for the probability of error detection (Ped) and the probability of false rejection (Pfr) of each TORCH’s panel marker.

## Results

We developed the quality planning for eight TORCH panel markers [Toxoplasma gondii (Toxo), Rubella, Cytomegalovirus (CMV) and Herpes Virus 1 (HSV1) and HSV2], and a total of 44,788 analyses were reviewed. The minimum acceptable concentration was obtained, finding an average of 20.8 ± 7.4% for the quality requirements for each marker. The imprecision analysis was assessed by intra-assay CV% for each infectious marker. In addition, an average imprecision of 3.69 ± 1.47% was found. Our results indicate that CMV had lower imprecision (2.3% for IgM and 2.6% for IgG) while Toxo had a 5% imprecision and 5.38% for Toxo IgM and IgG, respectively.

Quality planning of the TORCH panel allowed estimating the sigma value that ranged from 4 to 10, where rubella had the highest values (10 for IgM and 8 for IgG) while HSV2 had the lowest values (4 for IgM and 5 for IgG). These results appear in Table 1. Fifty percent of the tests had a sigma greater than 6 (average 7 ± 2 sigma).

**Table 1:** Baseline results for the TORCH panel tests evaluated with the sigma metric

TORCH panel	Abs	Cutt-off	Quality requirements^a^	Imprecision^b^	Sigma	∆SE
*Toxoplasma gondii*						
IgM	0.007	0.005	28.6	5	6	4.06
IgG	0.012	0.008	33.3	6.2	5	3.73
Rubella						
IgM	0.027	0.02	25.9	2.6	10	8.35
IgG	0.219	0.177	19.2	2.4	8	6.35
Cytomegalovirus						
IgM	0.054	0.047	13	2.3	6	3.99
IgG	0.022	0.019	13.6	2.6	5	3.59
Herpes simplex virus 1						
IgM	0.084	0.074	11.9	3	4	2.32
IgG	0.023	0.019	17.4	2.7	6	4.79
Herpes simplex virus 2						
IgM	0.055	0.045	18.2	4.6	4	2.30
IgG	0.015	0.011	26.7	5.5	5	3.20

*Abs* absorbance (optical density δ)

^a^In %

^b^In %CV

**Fig. 1:**
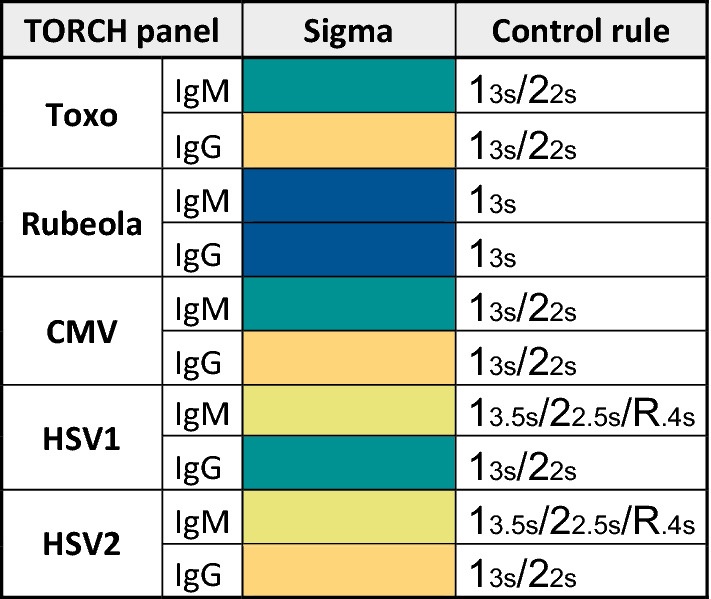
Westgard rules selected for TORCH panel control. For both IgM and IgG, sigma greater than 6 (blue box), sigma of 6 (green box) and sigma between 4 and 5 (yellow box) are shown

**Fig. 2:**
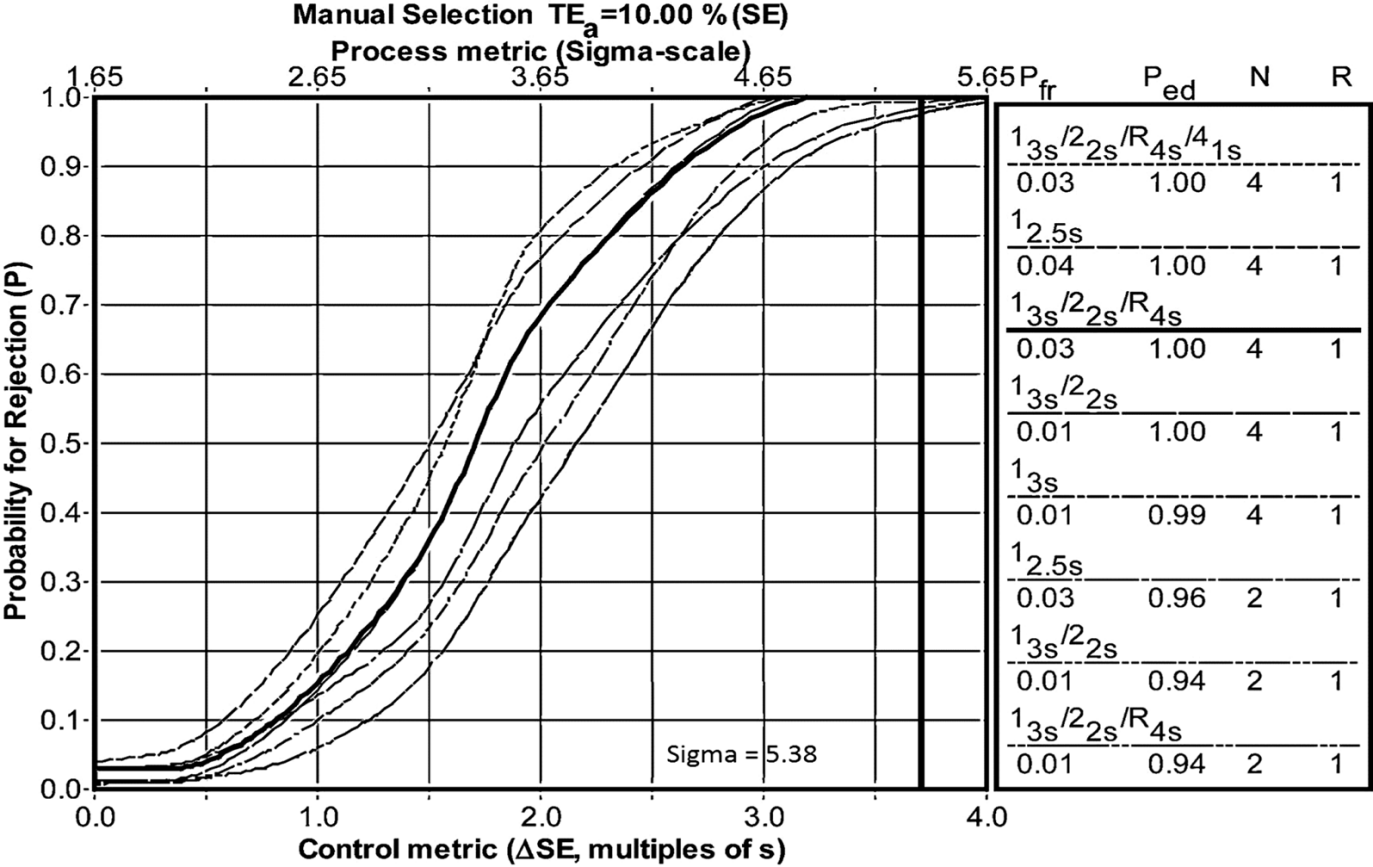
Sigma control chart for Toxoplasma IgG

The results shown in Fig. 1 reveal the selected control rules for the TORCH markers. For the two rubella markers, IgM of Toxo and CMV, and IgG of HSV1, control multi-rules were chosen as 13s/22s that provided a 100% probability of detecting a critical loss of assay sensitivity (Pfr  = 0.01). On the other hand, the IgM marker of HSV1 and HSV2 required a stricter multi-rule as 13.5s/22.5s/R.4s (Ped  = 1 and Pfr  = 0.03) to remain in control. None of the sigmas found invalidated the use of TORCH markers. The rule selection process is in Fig. 2.

## Discussion

This study of quality planning in qualitative tests suggest that 50% of markers from the TORCH panel had an optimal sigma (> 6) allow the selection and application of Westgard control rules in a reference laboratory in Peru.

The strengths of this study lie in being one of the first studies to apply the sigma metric to qualitative TORCH tests in Latin America. Also, this study used a qualitative test analysis algorithm that demonstrated an optimal level of sigma performance of the TORCH panel. Knowing the efficiency of these markers is crucial to ensure neonatal health.

In order to control a test, it is necessary to apply statistical control methods that allow the test to be maintained within typical values and to be able to demonstrate the alterations that diseases demand. Although this is the goal of quality control processes, planning is the pillar to organize all processes and ensure the quality of results [13, 14]. Modern practical concepts within analytical quality planning include using sigma metrics as a widely applicable method with a fast and simple calculation to organize quality axes in daily clinical practice.

Our findings demonstrated excellent performance for Rubella (> 6 sigma) and good performance for Toxo and CMV (5–< 6 sigma). These sigma results are substantially higher compared to two studies on thyroid hormones in the Indian population and transfusion viral infectious markers in the Mexican population that reported a sigma of  < 3 and  < 4, respectively [7, 8]. We recently demonstrated following the same algorithm optimal sigma in 2/7 infectious markers in a Peruvian blood bank [5]. Both previous results indicate the suitability of this quality planning method for qualitative tests.

Six sigma has demonstrated efficiency in the assessment of clinical laboratory assays using software or manually with multiple institutional benefits [15–17]. In low-income settings laboratories, the implementation of efficient quality programs is so necessary because in Peru [18], several public and private laboratories work daily without establishing their quality limits, putting at risk medical decisions based on clinical analysis.

Although laboratory medicine has been transcendental for Peruvian medicine [19], there is no organized program for global quality assurance. Even the National Institute of Quality (INACAL) does not ensure the standardization of its tests. Therefore, the overall quality of the results issued is still unknown. Evaluations under sigma metrics have an increasing impact on qualitative tests as a result of the studies and models that have gradually emerged in the last two decades [20]. Further studies are needed to scrutinize the quality assurance processes using sigma metrics in qualitative tests.

In this study, the selection of control rules has been multi-rule as it is set out with a high Ped to assess both systematic and random error. We have been most insidious with markers of sigmas of 4 for IgM of HSV1 and HSV2. Quality control, including the frequency of control and validation processes, the maintenance of equipment and staff training and participation in external and internal quality control programs are necessary to keep these markers within quality standards [14, 20, 21].

## Conclusion

Quality planning of the TORCH panel in the Peruvian population demonstrated optimal performance of half of the markers, including Toxoplasma, Rubella and CMV. Using six sigma multi-rule controls were selected for HSV1 and HSV2 that had 4–5 sigma to maintain quality. This study continues the roadmap for quality assurance of qualitative tests used in the clinical laboratory as routine tests; the development of quality planning processes for qualitative tests should be a commitment for all health care institutions.

## Limitation

The performance and sigma value of the TORCH panel could be affected by intra and inter-individual variations in the study subjects as the prevalence of infectious markers and the kind of users of the Suiza Lab in Lima, Peru. In addition, this quality planning was conducted on fourth-generation GEMINI XCR200 ELISA automated equipment; however, performance could vary with the CLIA test. Finally, due to its cross-sectional design, it was impossible to establish causal relationships between the sigma values of panel performance with some process features (i.e., pre-analytical errors, calibration frequency, participation in external quality programs).

## Data Availability

None.

## Abbreviations

TORCH: Toxoplasmosis, other infections, Rubella, Cytomegalovirus, and Herpes simplex virus
ELISA: Enzyme linked immunosorbent assay
CLIA: Chemiluminescence immunoassay
Toxo: Toxoplasma
CMV: Citomegalovirus
HSV1: Herpes simple virus 1
HSV2: Herpes simple virus 2
Ped: Probabilidad de detección de error
Pfr: Probabilidad de falso rechazo
